# On Lacerations of the Perineum

**Published:** 1874-03

**Authors:** Wm. Goodell


### ON LACERATIONS OF THE PERINEUM.

By Wm. Goodell, M.D.

*From the Philadelphia Medical and Surgical Reporter, Feb.* 21, 1874.

THE immediate closure of the rent in lacerations of the perineum ought by this time to be fully recognized by the profession as a very important means for the prevention of future mischief to the reproductive organs. As I have elsewhere shown (*Trans. of the State Med. Society of Pennsylvania, for* 1873), and here take the liberty of repeating, the loss of every fibre of muscle in the perineum entails a corresponding loss of power in the floor of the pelvis, and a consequent impairment of support to the reproductive organs. The sustaining power of the vaginal column depends upon the integrity of its perineal abutments. It is the tonicity of the vaginal walls, and the pelvic connections of the womb, that mainly keep it in place. These, in a case of torn perineum, may not at once yield, but will sooner or later; for air gains access to the womb, irritating and congesting it to such a degree that it ultimately prolapses from an acquired hypertrophy. Unless, therefore, the rent is simply cutaneous, or very slight indeed, it should not be left to nature. Further, it is far more rational to take advantage of the necessary confinement in bed after delivery, and to close the wound at once, while its surface is raw, and the maternal soft parts are comparatively numb and insensible, than to postpone the operation to a time when the woman shall be nursing, when the cicatrized flaps shall demand quite a formidable operation for their denudation, and when a special confinement in bed for two weeks or more will be needed.

My own method is, immediately after the delivery of the placenta, to pass deeply two or more wire sutures, securing each one by merely twisting its ends together. In bad rents, the first stitch is entered not quite half an inch below the lower angle of the wound, and about an inch from its margin. When the sphincter ani is torn, the cutaneous points of entrance and of exit of the first needle should then be nearly on a level with the lower margin of the anal orifice, and the suture should pass around the whole wound. This purses up the tissues from below upward, and secures complete coaptation. Enough opium must be given daily to keep the bowels quiet for a week.

In severe lacerations the woman's knees must be kept bound together for a week, and her urine drawn off for three or four days. On the third or fourth day, but not earlier, lest the process of immediate union should be interrupted, vaginal injections of weak solutions of carbolic acid, or of the permanganate of potassa, are made twice in the twenty-four hours. These soothe the parts, and correct the bad odor of the discharges. Without reference to any special time, the sutures are removed as fast as they become loose, usually from the seventh to the ninth day. On the eigth or tenth day a seidlitz powder, or one dessert-spoonful of castor oil, is given every four hours until an inclination to go to stool is urgent; then an injection is given in order to liquify the contents of the lower bowel. This method of uniting the parts, both in the immediate and in the secondary operation, after the cicatrized surfaces are denuded, I can warmly recommend, as I cannot recall but one case, and that a very unruly one of puerperal mania, in which there was failure in obtaining a very good union. It ought, however, to be stated, that in secondary operations superficial sutures should be placed between the deep ones, and that the latter should be clamped with perforated shot. In order, also, to pare each side of the rent with unerring uniformity, after freshening the surface of one side, its exact print in blood can be got on the other by pressing the nates together for an instant. A very troublesome symptom in these cases is flatus. If it does not yield to valerian, a gum catheter should be very carefully passed up into the rectum.

Many lacerations are, in my opinion, owing to the very common mistake of making so firm a pressure upon the perineum as to prevent it from undergoing an equable dilation. The portion thus compressed cannot take its share of the general tension, and the strain is thrown on the fourchette. Further, the pressure of the hand, by obstructing the free circulation of blood, impairs the vitality of the perineum. Bruised and benumbed, it is no longer a living tissue, capable of responding intelligently, so to speak, to the requirements of the occasion—when to repel, when to solicit, the advance of the head—and this nice point nature can very generally determine far better than the physician. Again, the word "*support*," as applied to the perineum, is a misnomer. No "support," in the ordinary acceptation of the word, is afforded to the perineum by direct pressure. If such a method ever accomplishes any good, it is by retarding the advance of the head, in other words, by *supporting* the head through the interposed perineum, and not by supporting the perineum itself. Why not, then, support the head by pressure directly applied to it, instead of through a medium which requires perfect freedom from all restraint in order to undergo the requisite and inevitable amount of dilatation? Finally, a majority of the advocates of "support" contend that it is most needed at the very moment of expulsion. But the woman, in the agony of the final throes, is very likely to jerk herself away from the hand of the accoucheur. Of course, then, the perineum, being abruptly released from counter-pressure, is more liable to yield to a strain suddenly sustained, for which its fibres are unprepared. Obstetric teachers recognize this danger, and in vivid language caution the student against it.

Although I believe that in the vast majority of labors the perineum does best when let alone, yet cases do undoubtedly arise which demand an intelligent assistance; nor can the line of demarkation be always drawn between natural and morbid conditions. Whenever the head in an occipito-anterior position is too much flexed, and the vertex bears on the perineal center, threatening perforation; whenever, in an occipito-posterior position, the head is too little flexed, the forceps are urgently needed. For cases of extreme rigidity, or of an undersized vulval opening, ether will be found a potent remedy. Apart from a direct and retarding pressure upon the presenting part itself, the only manual aid that I permit myself to render is as follows: Insert one or two fingers of the hand into the rectum, the woman lying indifferently on her side or on her back, and hook up and pull forward the sphincter ani towards the pubes. The thumb of the same hand is then to be placed upon the fœtal head, scrupulously avoiding all contact with the fourchette. For this method I claim the following advantages: (*a*) By pulling up the sphincter ani towards the pubes not only is nature imitated, which always dilates the anal orifice, but the perineum is brought forward without direct pressure, and its dilatation is diffused over its entire surface, causing a corresponding relaxation of the strain on the posterior commissure, in the line of its raphe. In addition, its muscular fibres are crowded up to, and consequently strengthen, the line of greatest tension; just as a prudent general hurries up reinforcements to the point of attack. (*b*) The same force which dilates the sphincter ani compels the occiput to hug the pubes, and favor extension, especially if the fingers in the rectum are hooked over the prominences of the fœtal face, or over the chin. (*c*) This aid is not liable to sudden interruption from the movements of the woman. (*d*) The thumb of this hand, together, if necessary, with the fingers of the free hand, can, by direct pressure upon the presenting part, restrain its too rapid advance, without exciting that reflex uterine action which is so frequently evoked by the irritation of contact with the perineum. (*e*) The circulation of the blood remains free; the nerves are not benumbed by a double pressure, and the perineum, therefore, continues in its natural condition, that of a living, elastic and sentient tissue. This method I have more fully described in an essay published in the *American Journal of the Medical Sciences*, January, 1871, p. 75. To it I beg leave to refer those of my readers who are interested in the subject of the management of the perineum during labor.

Misdirected traction on the after-coming head, viz., too much in a downward direction as the head is about to emerge, is very commonly followed by a very bad rent of the perineum. Even in head-presentations, requiring apparently but slight traction, the use of the forceps will often occasion a slight tear in the vagina, which the passage of the shoulders prolongs into the perineum. From too hurried a delivery, or from faulty traction, I have seen so many bad lacerations following the use of this instrument, even in practiced hands, that I can not withhold the opinion that, in the majority of cases, nature can accomplish the final delivery of the head through the soft parts much better than the physician. In the essay previously adverted to, I use the following language, which the riper experience of three years more has not induced me to change: "Delivery by the forceps, even in skillful hands, will often produce laceration; for the head is liable to be brought down too quickly on the unprepared soft parts, and it becomes a very nice point indeed to determine the exact moment when delivery may be ended with impunity. The cautious physician is liable to be caught, as it were, 'on the center.' He sees the perineum stretched out to a perilous thinness, and the fourchette almost cracking under the strain. In doubt whether the moment has arrived to raise the forceps-handles and turn out the head, or to depress them, and thus restrain its advance, he wavers, and in a twinkling the fibres part. On the other hand, the impatient physician is tempted to turn out the head before the parts are sufficiently dilated. Finally, what is still more frequent, at the last moment the physician's courage fails him, and he depresses the forceps-handles just as the head has begun to emerge; a course equally fatal to the integrity of the perineum." My advice is, therefore, that, other things being equal, as soon as the perineum is well dilated, the forceps should, as a rule, be removed, unless the blades are so firmly imbedded in the child's tissues that their withdrawal requires a force which might hasten the delivery of the head. This practice, if not so brilliant, will, I believe, in the long run be found much safer.

At the risk of becoming prosy on this subject, I wish to add my conviction that, through sentiments of delicacy, many lacerations of the perineum escape the notice of the physician. After the delivery of the placenta, he should, therefore, make it a rule to introduce the index-finger into the rectum, and the thumb into the vagina. By bringing them together he can estimate the thickness of the intervening tissue, and thus determine whether any extensive laceration has taken place. If a rent be discovered, he should decently inspect the parts. By daylight, this examination can usually be made without the knowledge of the patient. When candle-light is needed, he will be compelled either to make some excuse, or boldly explain his object.

